# High levels of alpha-gal with large variation in the salivary glands of lone star ticks fed on human blood

**DOI:** 10.1038/s41598-023-48437-2

**Published:** 2023-12-04

**Authors:** L. Paulina Maldonado-Ruiz, Kathryn E. Reif, Anuradha Ghosh, Stephanie Foré, Rachel L. Johnson, Yoonseong Park

**Affiliations:** 1 Department of Entomology, Kansas State University, Manhattan, KS 66506 USA; 2 Diagnostic Medicine/Pathobiology, Kansas State University, Manhattan, KS 66506 USA; 3 Department of Biology, Pittsburg State University, Pittsburg, KS 66762 USA; 4 Department of Biology, Truman State University, Kirksville, MO 63501 USA

**Keywords:** Entomology, Zoology

## Abstract

Tick bites, associated with the secretion of tick saliva containing the xenoglycan galactose-alpha-1, 3-galactose (alpha-gal or aGal), are recognized as the causal factors of alpha-Gal syndrome (AGS; or red meat allergy) in humans. AGS occurs after the increased production of IgE antibodies against aGal, which is found in most mammalian cells, except for the Old World monkey and humans. The aGal sensitization event has been linked to an initial tick bite, followed by consumption of red meat containing the aGal glycan, which triggers the onset of the allergic response resulting in urticaria, anaphylaxis, or even death. In North America, the lone star tick, *Amblyomma americanum*, has been identified as the main culprit for AGS. However, only a subset of the human population exposed to lone star tick bites develops AGS. This suggests the presence of unidentified variables associated with the sensitization event. To evaluate the quantitative variations of the aGal in ticks, we evaluated the differences in aGal levels in different strains of *A. americanum* ticks partially fed on different blood sources using an artificial feeding system and animal hosts. We found significantly higher aGal levels in the female ticks fed on human blood than those fed on the blood of other mammals with large variations among different tick populations and individuals. We propose that host-specific genetic components in the *A. americanum* ticks are involved in the production of high aGal epitope in the tick saliva, which provides a part of the explanation for the variables associated with the AGS sensitization event of the tick bite.

## Introduction

Alpha-gal syndrome (AGS, or red-meat allergy) is often a life-long condition characterized by allergic reactions to red meat or other products containing the xenoglycan galactose-alpha-1, 3-galactose (aGal)^1,2^. Allergic reactions to other aGal-containing products and in xenotransplantation have been reported, such as; anaphylactic responses to cetuximab, a murine-derived monoclonal anti-cancer antibody containing aGal, and the rejection of transplanted porcine aortic/cardiac valves^1–5^. AGS is triggered by the enhanced production of IgE antibodies against aGal commonly present in most mammalian tissues, with the exceptions of Old World monkeys and humans. The pseudogenization of the alpha-1,3-galactosyltransferase gene in these lineages allowed the development of immune defenses against pathogenic organisms carrying aGal^3,6–8^ at the expense of a hyper-reaction through IgE present in AGS.

In the United States, bites of the lone star tick (*Amblyomma americanum*), associated with injection of tick saliva containing aGal^9–11^, are thought to be the main AGS sensitizing event. A growing number of cases in the Southeast and Midwest regions of the United States, which overlap with the endemic areas of the lone star tick, have been reported^3,12^. This was demonstrated in the alpha-galactosyltransferase knockout mouse model system (αGT-KO) lacking aGal^13,14^. While this condition has been associated to tick bites from different species worldwide, including *Ixodes* spp. in Europe and Australia and *Hemaphysalis longicornis* in Asia^15–18^, the sources of the aGal sensitizer in tick salivary glands (SG) and SG secretions are not fully explored.

Interestingly, AGS occurs in only a small subgroup of the human population experiencing tick bites, indicating that only a subpopulation of the human exposed to tick bites is vulnerable to the allergic response or only a subpopulation of ticks is responsible for triggering the sensitization event. Previous studies have shown that salivary glands of *A. americanum* contain a large amount of host-associated proteins, including *N*-glycosylated proteins containing aGal^19,20^. In addition, we have previously shown that the SG of *A. americanum* ticks fed on bovine blood contained aGal glycosylated proteins and other cross-reactive carbohydrates^10^. In this study, we report that ticks fed on human blood produce significantly higher levels of aGal than those fed on the blood of other mammals, with large variations among individual ticks and different populations. We propose that this variation may contribute to why AGS occurs only in a subpopulation of humans experiencing tick bites.

## Results

### High aGal levels in the tick SG fed on human blood

**Figure 1:**
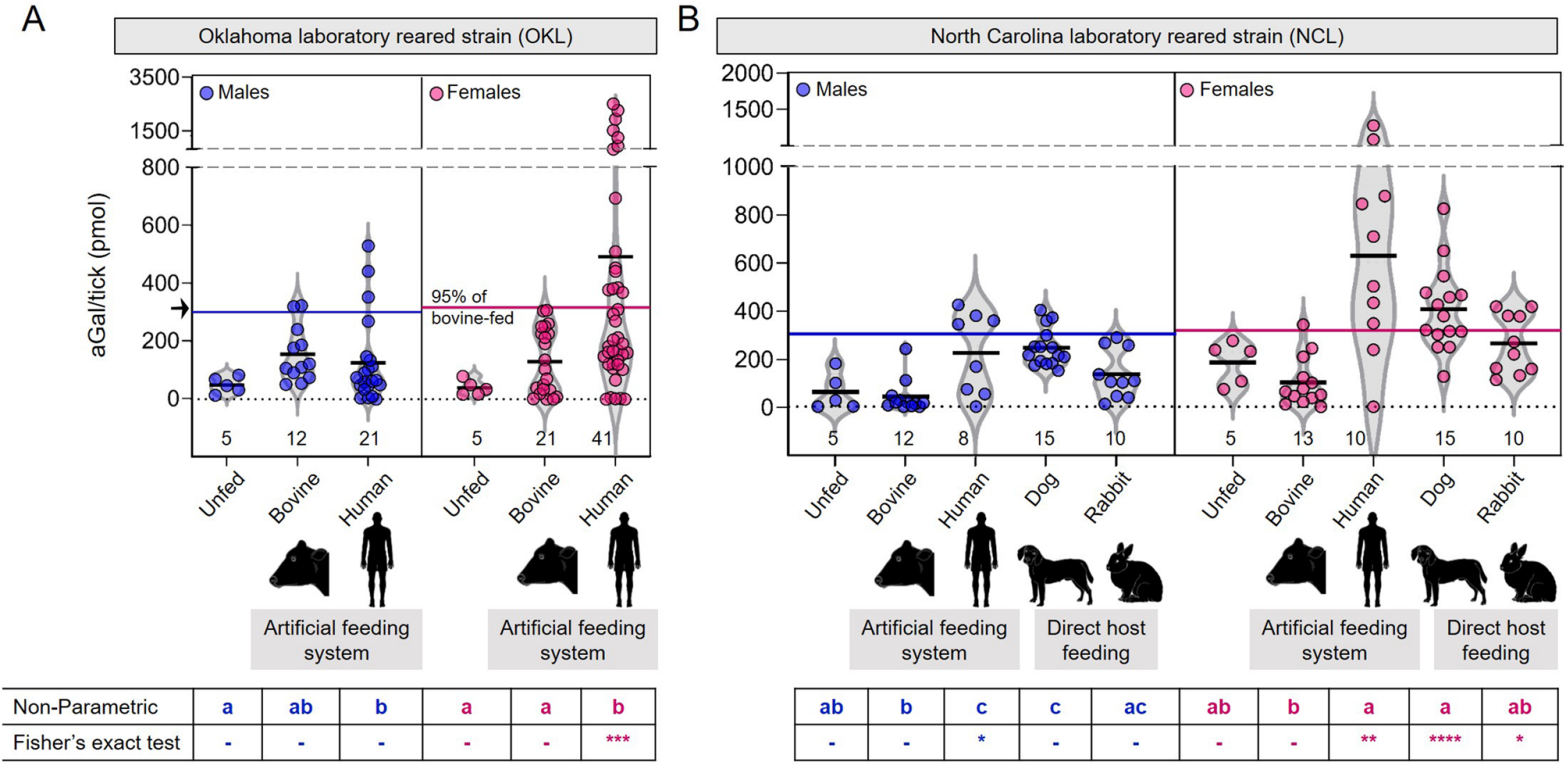
Alpha-Gal levels in SG ticks fed on different sources. (**A**) OKL ticks fed on bovine blood and human blood. (**B**) NCL ticks fed on artificial feeding system or directly on animal hosts. Alphabet assignment for significance was conducted using an ANOVA test (parametric and non-parametric, Kruskal–Wallis) at p < 0.05. The ANOVA multiple comparisons tests were conducted within sex group (males in blue, females in magenta). Fisher’s exact test was conducted by comparing the frequency of individuals outside the 95% normal distribution cutoff for the OKL-bovine-fed group (301.2 pmol/tick for the males and 317.6 pmol for the females). Figure was created with GraphPad Prism 9 and MindtheGraph.com (accessed on November 2023).

Ticks partially fed on human blood produced significantly higher levels of aGal in their salivary glands than those fed on the blood of other mammals, with a large variation among individual ticks. Oklahoma laboratory strain (OKL) females partially fed on human blood through an artificial membrane showed significantly higher aGal levels in salivary glands compared to those fed on bovine blood (492 vs. 129 pmol/tick on average) with a large variation at the individual tick level (Fig. 1A). Of the female ticks partially fed on human blood, 39% individuals showed significantly higher levels of aGal compared to those fed on bovine blood (the 95% cutoff in bovine blood-fed ticks 318 pmol, Fisher’s exact test *p* = 0.0005). A few ticks showed noticeably high levels of aGal, up to 3325 pmol/tick. Male ticks fed on human blood also showed a tendency to higher levels of aGal than those fed on bovine blood (Fig. 1A). In an expanded experiment with the strain from North Carolina (NCL), similar patterns with significantly higher aGal levels in human blood-fed ticks compared to bovine blood-fed ticks (627 vs. 101 pmol/tick) with a large individual variation ranging 0–1262 pmol/tick (Fig. 1B). The NCL strain directly fed on other hosts, dogs or rabbits, showed lower levels of aGal compared to feeding on human blood, while the ticks fed on dogs showed moderately high levels of aGal (Fig. 1B).

### High variations in the aGal levels in the tick SG among different geographical populations

**Figure 2:**
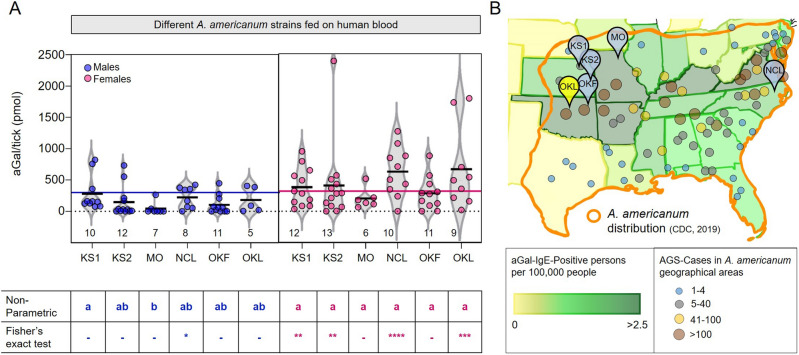
Alpha-Gal levels in SG of ticks fed on human blood by tick location and during feeding. (**A**) Field collected: KS1, KS2, MO, OKF, and laboratory-reared: NCL, and OKL, and table depicting statistical significance in the Kruskal–Wallis one-way-ANOVA multiple comparisons test and the Fisher’s exact test (significance *p* = 0.05). (**B**) Map of the Midwest and Southeast regions of the United States indicating tick geographical locations used in this study, previous reports of alpha-gal IgE-positive persons and AGS cases^12,32^, and *A. americanum* distribution. The map was constructed manually using previously published data^12,32,33^ using Adobe Inc., 2019. Adobe Illustrator (available at https://www.adobe.com/products/illustrator.html) and edited in Adobe Fresco (available at https://www.adobe.com/products/fresco.html).

We tested aGal levels of the field-collected ticks from different geographical locations, after being partially fed on human blood (Fig. 2A). The experimental cohort in the comparisons among geographically different populations included repeated tests of the tick laboratory strains, OKL and NCL, which have been maintained with a periodical introduction of respective local field tick populations. Field-collected ticks from two different locations in Kansas (KS1 and KS2), and one each from Oklahoma (OKF) and Missouri (MO), were tested (Fig. 2B). Fisher’s exact test, testing the differences of the frequencies that are higher than those of bovine blood-fed ticks (> 318 pmol), found KS1, KS2, NCL, and OKL females have significantly higher frequencies of high aGal ticks although this non-normally distributed data set in nonparametric ANOVA test found only male MO population different compared to others (Fig. 2A).

### Alpha-gal levels in tick SG increase during human blood feeding

**Figure 3:**
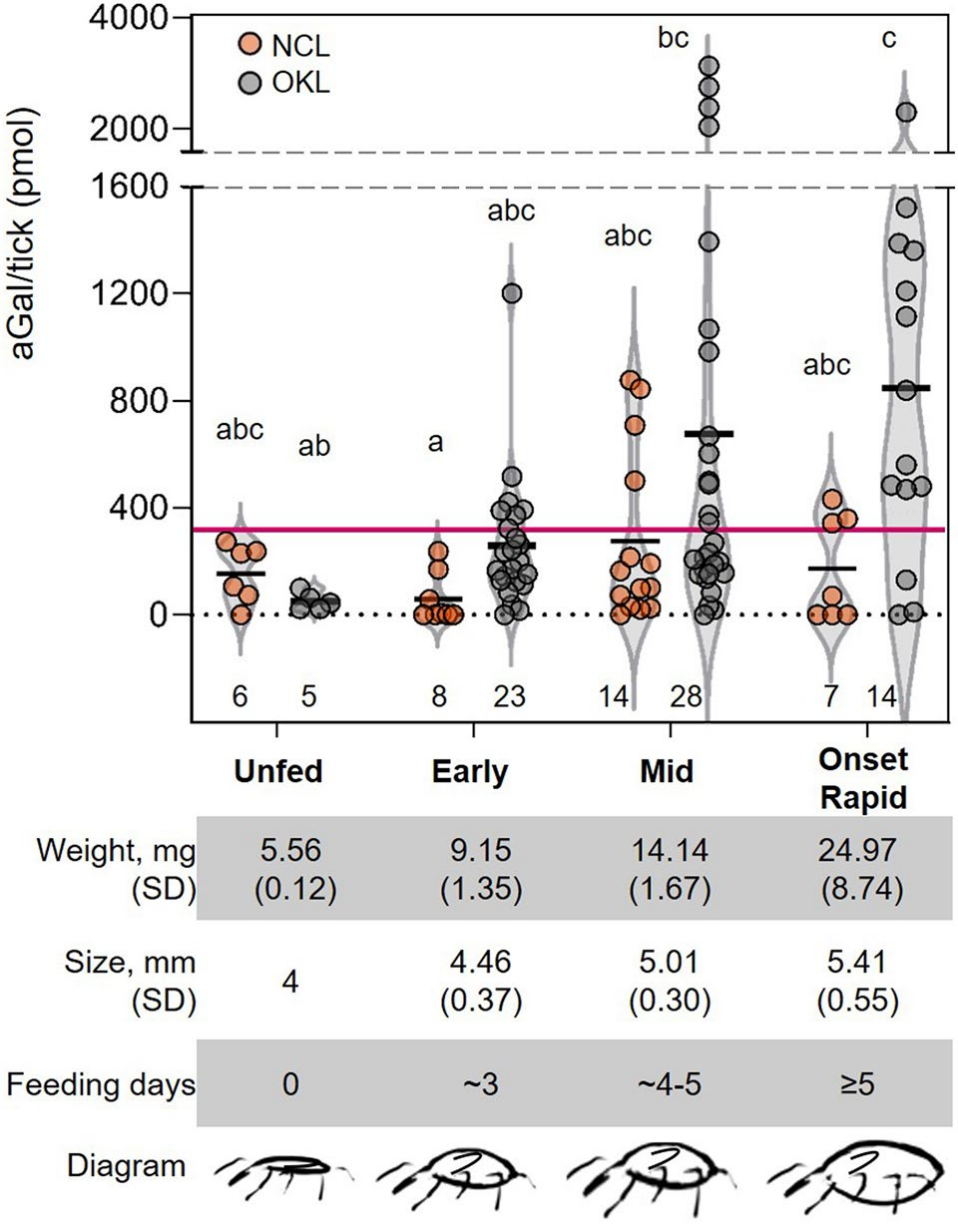
Alpha-gal levels in SG of ticks fed on human blood during feeding. Alpha-gal levels of Lab-reared tick strains during the feeding stage, stages were classified according to feeding time, weight, and size, with a schematic representation of the increase in size during the course of feeding. The alphabets show significant differences at *p* = 0.05 in the Kruskal–Wallis one-way-ANOVA test. Tick illustrations were created in Adobe Fresco (available at https://www.adobe.com/products/fresco.html).

To measure aGal level changes dependent on the feeding stage, we classified the tick feeding stage based on size, weight, and days of feeding. Ticks were classified as, (1) early feeding phase, less than 3 days of feeding and weight range from 4.5 to10.9 mg; (2) mid-feeding phase, 4 to 5 days of feeding and from 11 to14.9 mg; and (3) onset of rapid engorgement phase, ≥ 5 days of feeding and above 15 mg. A tendency of increased aGal levels in longer feeding was noticed with the presence of ticks exhibiting remarkably high aGal levels between 4 and 5 days of feeding (Fig. 3). The multiple comparisons ANOVA test showed only one significant difference between the OKL unfed and the onset of rapid engorgement stages (Fig. 3).

## Discussion

The variables that link lone star tick bites and the occurrence of AGS in humans likely involve complex factors. The levels of the aGal glycan in the salivary glands of lone star ticks could play a crucial role in the sensitization process of AGS. The high levels of aGal found in several ticks fed on human blood in this study could be a factor that contributes to AGS sensitization. The tick salivary components decorated with aGal were previously hypothesized as a mechanism of molecular mimicry in the ticks on non-human mammalian hosts for evasion of host immune systems. The high aGal level in ticks feeding on humans is contradictory in this sense, as aGal in the tick saliva would enhance host defenses by the host antibodies against aGal. Therefore, the role of high aGal level in the ticks feeding on humans, an incidental host, is yet enigmatic. Tick feeding on avian hosts, which also lacks aGal, could be investigated to understand the roles and the mechanisms involved in high aGal levels in the tick SG.

Moderately high levels of aGal were also observed in the ticks fed directly on the dogs, which was statistically higher than those in ticks fed on bovine blood (Fig. 1B). Interestingly, dogs are known to have relatively high levels of IgG against aGal, which is also induced by tick bites, despite the presence of endogenous aGal^21^. Clinical adverse food reactions to meats and dairy products have been frequently reported in dogs^22^. The possibility of the link to aGal in meat allergy in dogs needs to be further investigated.

This study also supports the previous study rejecting the aGal transmission hypothesis in AGS where the source of aGal in tick saliva was hypothesized from prior feeding on non-human mammalian hosts^14^. In our previous study using the mouse model with tick infestation, we observed that aGal levels in the mouse-fed ticks were low (Fig. S1) (see Supplementary Information) with highly varying levels of mouse antibodies specific to aGal^14^. While the alpha-1,3-galactosyltransferase orthologue gene has not been detected in ticks^23^, earlier research has indicated the potential involvement of other tick galactosyltransferases in the decoration of saliva with aGal. Multiple copies of genes such as alpha-1,4-galactosyltransferase, beta-1,3-galactosyltransferase, and alpha-1,4-galactosyltransferase have been reported in ticks^10,23,24^ and a number of those expressed in the salivary glands were suggested to have alpha-1,3-galactosyltransferase activity.

Another interesting aspect of tick bites causing AGS was the possible involvement of tick microbiomes including endosymbionts. A recent study proposed that an invasion of *Francisella*-like endosymbiont (FLE) replacing *Coxiella-like* endosymbiont (CLE) in *A. americanum* is associated with AGS^25^, although the genome sequences of the FLE, CLE, and another common endosymbiont, *Rickettsia amblyommatis*, do not contain bacterial alpha-1,3-galactosyltransferase gene in our search on the genome sequences. In addition to endosymbionts, other tick gut microbes may be involved, as a study in human gut microbiome found *Enterobacteriaceae* with alpha-1,3-galactosyltransferase genes. *Enterobacteriaceae* is a keystone taxonomic group found in *Ixodes ricinus* and also in *I. scapularis *^26–28^. However, our study is limited by the lack of microbiome characterization for the same samples determined for the aGal levels. Nevertheless, the mechanisms involved in the increased production of aGal proteins with the roles of tick microbiomes in AGS need to be further investigated.

Our results also show that the aGal levels in tick SG fed on human blood increase significantly over the course of feeding, with an increase seen as early as day 3 of tick feeding in the OKL tick strain (Fig. 3). Feedings of hard ticks are characterized broadly in two phases in the natural host; a slow/early feeding phase (phase I) in the first several days, and a rapid engorgement phase (phase II), in which significant body enlargement occurs^29,30^. Phase I is thought to be where the majority of tick salivary secretions occur, in preparation for feeding, and thus pathogen transmission can occur^31^. While the immediate correlation and the clinical relevance of high aGal levels in the early feeding phase are unknown, the early aGal synthesis may pose an additional risk factor for AGS in human feeding. Nevertheless, our findings suggest that early tick removal would be a preventative measure to reduce the risk of AGS.

Overall, we found that ticks that fed on human blood produced markedly higher levels of aGal compared to those that fed on blood from other mammals, with large variations among different geographical populations and individual ticks. The aGal variation found in this study likely involves differences in the tick genetic factors and may provide further knowledge for understanding the AGS sensitization process with further studies testing other blood sources lacking aGal, such as avian blood, and additional sampling of field tick populations can further support our findings. We propose that the high levels of aGal and the large variation in the levels of tick salivary aGal, the sensitizer for AGS, are factors contributing to the variation in the manifestation of AGS. Nevertheless, further understanding of the mechanisms and the genetics of high aGal will allow us to predict, prevent, and mitigate AGS.

## Methods

### Ticks and tick feeding

In our previous study, we observed lower levels of aGal in the ticks fed on mice than those fed on bovine blood in male ticks. This observation motivated us to further investigate the variations of aGal levels among different ticks. Unfed (naïve) adult male and female *A. americanum* ticks obtained from rearing laboratories or field collections were kept at room temperature with > 95% relative humidity (RH) until feeding. Ticks used in this study were obtained all in 2022 from regions with known reported cases of AGS. Laboratory-reared *A. americanum* ticks were obtained from Oklahoma State University lab rearing facility (OKL) and North Carolina tick rearing EctoServices, Inc. (NCL). For field-sourced populations, questing *A. americanum* were collected from northeastern Kansas (Manhattan, Konza Prairie Biological Research Station; Riley County, KS1), southeastern Kansas (Frontenac Wilderness Park; Crawford County, KS2), Oklahoma (Bartlesville, Washington County; OKF) and Missouri (Kirksville Union Ridge Conservation Area; Adair County, MO) by flagging. Ticks were fed for 3–5 days using defibrinated bovine blood (Hemostat Laboratories, CA, USA) or defibrinated human blood (BioIVT, NY, USA) using a modified artificial feeding system^10,14^ with a silicon membrane. NCL ticks fed on dogs were collected 72 h (3 days) post-infestation (IACUC# 4677). Rabbit-fed NCL ticks were directly purchased from EctoServices, Inc. after feeding on rabbits for 48–72 h (2–3 days).

### Tick feeding categorization and dissection

After ticks were removed from the host or artificial membrane, tick weight was recorded and tick length was measured from the tip of the hypostome to the base of the alloscutum. Based on size, weight, and days of feeding ticks were classified as; (1) early feeding phase (less than 3 days of feeding and weight range from 4.5 to 10.9 mg); (2) mid-feeding phase (4 to 5 days of feeding and from 11 to 14.9 mg); and (3) onset of rapid engorgement phase (≥ 5 days of feeding and above 15 mg). Feeding phases defined in this study as early and mid are comparable to the previously described tick feeding phase 1 (up to 4 days of feeding) and our onset of rapid engorgement phase corresponds to the initial portion of feeding phase 2, described in *Ixodes ricinus* ticks^30^. Tick salivary glands (SG) were dissected immediately after removal from feeding chambers for OKL and NCL strains. Ticks fed on dogs were dissected within 12 h after detachment from the host. Those fed on rabbits in EctoServices, North Carolina were dissected within 24 after the detachment due to the delivery to Kansas by express mail. Half of the SG pair was used directly for protein extraction while the other half was stored at − 80 °C for future studies.

### Western blot of tick salivary glands (SG)

Protein from half of the SG pair from single individual ticks was extracted using the protein extraction reagent T-per™ (Thermo Scientific, MA, USA). An aliquot was loaded for sodium dodecyl-sulfate polyacrylamide gel electrophoresis (SDS-PAGE) at ~ 5–10 μg of protein per lane using a Mini-PROTEAN® TGX Stain-Free™ gel (Bio-Rad, CA, USA), and Precision Plus Protein™ Unstained standard (10–250 kDa) was used as a protein size marker (Bio–Rad). Gels were transferred to a ready-to-use pre-wetted polyvinylidene fluoride (PVDF) membrane (Bio-Rad, USA) using a Trans-Blot® Turbo™ Transfer System (Bio-Rad). Western blot for alpha-gal was conducted in an eZWestTM automated western blotting device (GenScript, NJ, USA), using 0.1% inulin as a blocking buffer. Mouse IgM monoclonal antibody against the aGal epitope (M86) was used as the primary antibody (Enzo Life Sciences, NY, USA) at 1:50, and goat-anti-mouse IgM conjugated to HRP was used as the secondary antibody at 1:1000 (Novus Biologicals, CO, USA). Antibodies were eluted in the eZWest diluent following the manufacturer’s instructions. Enhanced chemiluminescence (ECL) substrate was used for the visualization of immunoreactive bands (Bio-Rad, CA, USA). Images were captured using the C-Digit® Blot Scanner and immunoreactive bands were quantified using the Image Studio Digits software (Li-Cor Biosciences, NE, USA). Alpha-gal quantification was conducted using a standard, human serum albumin conjugated to aGal (aGal-HSA) at 0.2 µg/well, equal to 60 pmol of aGal per lane.

### Statistical analysis

We used a non-parametric one-way ANOVA test (Kruskal–Wallis rank test with multiple comparisons) with a significance level of 0.05 (p < 0.05). Multiple comparisons were made within the sex group. Since the data sets were highly skewed due to the small number of extremely high-value samples, the data were also analyzed by Fisher’s exact tests for the frequencies of the individual ticks that are higher than the 95% range of the ticks fed on bovine blood. A Fisher’s exact test with a significance level of 0.05 (p < 0.05) was conducted to evaluate the group differences based on the 95% normal distribution cutoff for the OKL-bovine-fed group, which was 301 pmol for the males and 318 pmol for the females. We constructed contingency tables using the frequencies of individuals from the cutoff against the frequencies for each group outside the cutoff for males and females separately. Data analyses and figures were constructed in OriginPro 2022b (9.7.5.184) and GraphPad Prism version 9.2.0 for Windows (GraphPad Software, San Diego, CA, USA).

### Ethical considerations statement

Beagle dogs used in this study were housed in an AAALAC-approved animal facility within the College of Veterinary Medicine at Kansas State University under the supervision of Kathryn E. Reif. All animal protocols were approved and compliant with the guidelines of/by the Institutional Animal Care and Use Committee (IACUC) (IACUC #4677). The human blood samples used in this study were purchased directly from BioIVT (NY, USA), which specializes in obtaining biospecimens for research purposes. Blood samples are collected by BioIVT, from consented donors under IRB-approved protocols. As such, limited information about the donors is accessible for researchers conducting the studies, with the exception of age, sex, and race unless otherwise requested. This study is reported in accordance with ARRIVE guidelines for animal research (https://arriveguidelines.org).

### Supplementary Information

Supplementary Figure S1.

## Data Availability

The data supporting this study’s findings are available from the corresponding author upon request.
