# Supplementary material for: High levels of alpha-gal with large variation in the salivary glands of lone star ticks fed on human blood
Source: Sci Rep. 2023 Dec 4;13:21409. doi: 10.1038/s41598-023-48437-2 (PMC10695944; doi:10.1038/s41598-023-48437-2)
Supplement: Supplementary file 1 — Supplementary Figure S1. [file 41598_2023_48437_MOESM1_ESM.docx]

**Supporting Information for:**

**High levels of alpha-gal with large variation in the salivary glands of lone star ticks fed on human blood**

L. Paulina Maldonado-Ruiz^1^, Kathryn E. Reif^2^, Anuradha Ghosh^3^, Stephanie Foré^4^, Rachel L. Johnson^1^ and Yoonseong Park^1^*

**
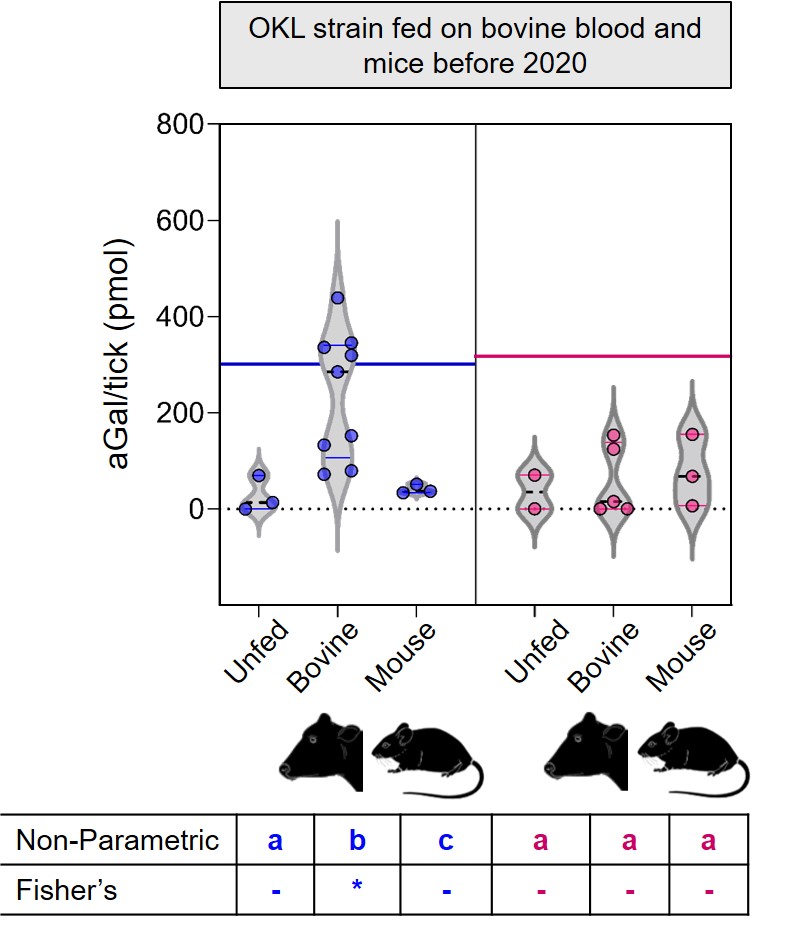
**

**Fig. S1. Alpha-Gal levels in the salivary glands of ticks that started feeding on bovine blood and finished feeding on mice for the experiments performed before 2020.** Note that the ticks were Oklahoma laboratory strain (OKL) before the yearly infusion of local populations after 2020. OKL ticks fed on bovine blood using the artificial feeding system and fed directly on C57BL/6 mice (from our previous study). Letter assignment for significance was conducted using an ANOVA test (non-parametric Kruskal-Wallis) at p<0.05. The ANOVA multiple comparisons test was conducted within the sex group (males in blue, females in magenta). Fisher’s exact test was conducted by comparing the frequency of individuals outside the 95% normal distribution cutoff for the OKL-bovine-fed group (301.2 pmol/tick for the males and 317.6 pmol/tick for the females) for each group. Figure was created with GraphPad Prism 9 and MindtheGraph.com (accessed on November 2023).
